# Optimal stereotactic body radiotherapy dosage for hepatocellular carcinoma: a multicenter study

**DOI:** 10.1186/s13014-021-01778-6

**Published:** 2021-04-21

**Authors:** Ting-Shi Su, Qiu-Hua Liu, Xiao-Fei Zhu, Ping Liang, Shi-Xiong Liang, Lin Lai, Ying Zhou, Yong Huang, Tao Cheng, Le-Qun Li

**Affiliations:** 1 Department of Radiation Oncology, Guangxi Medical University Cancer Hospital, Nanning, 530001 Guangxi Zhuang Autonomous Region China; 2 Department of Radiation Oncology, Rui Kang Hospital, Guangxi Traditional Chinese Medical University, Nanning, 530001 Guangxi Zhuang Autonomous Region China; 3 Department of Radiation Oncology, Changhai Hospital Affiliated To Navy Medical University, Shanghai, China; 4 Department of Hepatobiliary Surgery, Guangxi Medical University Cancer Hospital, Nanning, 530021 Guangxi Zhuang Autonomous Region China

**Keywords:** Hepatocellular carcinoma, Radiotherapy dosage, Stereotactic body radiotherapy, Survival rate

## Abstract

**Background:**

The optimal dose and fractionation scheme of stereotactic body radiation therapy (SBRT) for hepatocellular carcinoma (HCC) remains unclear due to different tolerated liver volumes and degrees of cirrhosis. In this study, we aimed to verify the dose-survival relationship to optimize dose selection for treatment of HCC.

**Methods:**

This multicenter retrospective study included 602 patients with HCC, treated with SBRT between January 2011 and March 2017. The SBRT dosage was classified into high dose, moderate dose, and low dose levels: SaRT (BED_10_ ≥ 100 Gy), SbRT (EQD_2_ > 74 Gy to BED_10_ < 100 Gy), and ScRT (EQD_2_ < 74 Gy). Overall survival (OS), progression-free survival (PFS), local control (LC), and intrahepatic control (IC) were evaluated in univariable and multivariable analyses.

**Results:**

The median tumor size was 5.6 cm (interquartile range [IQR] 1.1–21.0 cm). The median follow-up time was 50.0 months (IQR 6–100 months). High radiotherapy dose correlated with better outcomes. After classifying into the SaRT, SbRT, and ScRT groups, three notably different curves were obtained for long-term post-SBRT survival and intrahepatic control. On multivariate analysis, higher radiation dose was associated with improved OS, PFS, and intrahepatic control.

**Conclusions:**

If tolerated by normal tissue, we recommend SaRT (BED_10_ ≥ 100 Gy) as a first-line ablative dose or SbRT (EQD_2_ ≥ 74 Gy) as a second-line radical dose. Otherwise, ScRT (EQD_2_ < 74 Gy) is recommended as palliative irradiation.

**Supplementary Information:**

The online version contains supplementary material available at 10.1186/s13014-021-01778-6.

## Background

Hepatocellular carcinoma (HCC) is highly prevalent in many Asian countries and accounts for nearly 80% of HCC cases worldwide. In China, HCC is the second most common cause of cancer-related deaths and the fourth most commonly diagnosed cancer among men [1]. HCC is resectable in only 10–40% of newly diagnosed patients. Liver resection, transplantation, percutaneous ethanol injection, or radiofrequency ablation (RFA) are the standard treatments for early-stage HCC [2].

The use of external beam radiation therapy (RT) [3], specifically including stereotactic body radiation therapy (SBRT), is increasing in popularity of treatment for HCC [4–11]. It is commonly recommended as an alternative treatment in medically inoperable patients, as a result of its rapid adoption in clinical practice worldwide [12–14]. SBRT for primary HCC provides high rates of durable local control (89–100%) [8, 15–18], but there is no clear evidence of a dose-survival relationship for the commonly used radiation therapy schedules. Increasing radiotherapy dose was associated with improved overall survival in patients treated with SBRT for stage I non-small-cell lung cancer [19–21]. However, the optimal dose and fractionation scheme of SBRT for HCC remains unclear because primary HCCs tend to be associated with different degrees of cirrhosis and tolerated liver volumes. In a previous retrospective study of SBRT for 127 patients with HCCs that were > 5 cm, we preliminarily found that higher biologically effective dose (BED_10_) and equivalent dose in 2 Gy fractions (EQD_2_) was associated with better survival [22]. In another prior prospective study, we built normal tissue complication probability models and nomograms for radiation-induced hepatic toxicity to obtain individual liver constraints for HCC patient [23]. In current study, we aimed to verify the dose-survival relationship to optimize dose selection for treatment of HCC.

## Methods

### Study design and patients

This was a multicenter retrospective study of patients with HCC who underwent SBRT in China between January 2011 and March 2017. HCC diagnosis was established based on histopathology or according to the clinical criteria for diagnosis of HCC [13]. The eligibility criteria were as follows: primary or recurrent/residual HCC patients, who were medically inoperable or refused to undergo surgery and radiofrequency ablative therapy, treated with SBRT. The exclusion criteria were as follows: (a) prior history of abdominal conventional radiotherapy, (b) intrahepatic cholangiocellular carcinoma, (c) gallbladder metastases, and/or (d) liver metastases, (e) patients with incomplete data and lost to follow-up.

### Stereotactic body radiation therapy

Briefly, the patients were immobilized with a customized external vacuum-type. All patients were treated using the CyberKnife system (Accuray Incorporated, Sunnyvale, CA, USA), with 6 Mv photons. Three or four gold markers were inserted into the surrounding area of the tumor or into tumor tissue. Gross tumor volume was delineated as the visible tumor. Planning target volume was established as a 0–5 mm expansion of the GTV. No internal target volume was created because tracking was used. A dose of 28–55 Gy was administered in 1–6 fractions on consecutive days at the 50–85% isodose line that covered at least 97% of the planning target volume. Total doses and fractionation schedules were chosen according to size and dose-volume constraints of the organs at risk. The SBRT technique used has been previously described [5, 17, 22–24]

### Response evaluation and follow-up

Patients were re-evaluated 1 month after SBRT and every 3–6 months thereafter. In addition, contrast-enhanced CT or/and MRI were performed at each follow-up visit. The Modified RECIST Response Evaluation Criteria in Solid Tumors (mRECIST) guideline was used to evaluate the response of the tumor [25]. The laboratory examinations assessed levels of aspartate transaminase (AST), alanine transaminase (ALT), prothrombin time (PT), levels of albumin, total bilirubin, alpha fetoprotein (AFP).

### Calculated values

BED_10_ and EQD_2_ were assumed at an α/β ratio of 10, for rapidly proliferating tumor cells. EQD was calculated as: d × n{(α/β + d)/(α/β + dx)}; BED was calculated as: d × n{1 + d/(α/β)}; (d = dose, n = fraction and dx = 2). Based on our previous studies [22, 23], the SBRT dosage was classified into high dose, moderate dose, and low dose levels: SaRT (BED_10_ ≥ 100 Gy), SbRT (EQD_2_ > 74 Gy to BED_10_ < 100 Gy), and ScRT (EQD_2_ < 74 Gy).

### Statistical analysis

Overall survival (OS), progression-free survival (PFS) incidence of local recurrence (LC), and incidence of intrahepatic recurrence (IC) rates were estimated using the Kaplan–Meier method and compared between groups using the log-rank test. Cumulative OS was calculated starting from the date of the first treatment until the date of the final follow-up or death. Cumulative PFS was calculated starting from the date of the first treatment until the date of recurrence or progression or death. LC was calculated starting from the date of the first treatment until the date of local recurrence or progression. IC was calculated starting from the date of the first treatment until the date of intrahepatic recurrence or progression.

Additionally, variables without associations between each other were analyzed by chi-squared/Mann–Whitney-tests. We use univariate with significant value (*P* < 0.05) to identify non-associated predictive variables that contribute towards the final multivariate. For categorical variables, the Pearson's chi-squared test was used. Kruskal–Wallis test was used to analyze continuous variables.

All statistical analyses were performed using R version 4.0.2 (2020-06-22) software. *P* < 0.05 was considered statistically significant.

## Results

### Baseline characteristics

A total of 602 HCC patients with complete information were included in this study. All patients were classified into three groups according to SBRT dosage: SaRT (n =259), SbRT (n = 163), and ScRT (n = 180). The demographic and clinical characteristics of the patients and their treatment are summarized in Table 1. We observed strong associations between RT dose/fractionation and other prognostic factors, including BCLC class, tumor size, and ALBI grade. In general, patients with small tumors, BCLC stage A, and/or low ALBI score received higher RT doses, whereas those with larger tumors, BCLC B, C, D, and/or higher ALBI score received lower RT doses.

**Table 1:** Patient and treatment characteristics for different dose groups

Factor	Level	SaRT	SbRT	ScRT	*P* value
N		259	163	180	
Gender	Female	38 (14.7%)	23 (14.1%)	24 (13.3%)	0.92
	Male	221 (85.3%)	140 (85.9%)	156 (86.7%)	
Age, median (IQR)		54 (45, 64)	55 (45, 63)	51 (44, 58.5)	0.030
Age ≥ 60	No	164 (63.3%)	105 (64.4%)	137 (76.1%)	0.012
	Yes	95 (36.7%)	58 (35.6%)	43 (23.9%)	
HBV	Positive	188 (72.6%)	115 (70.6%)	123 (68.3%)	0.17
	Negative	33 (12.7%)	25 (15.3%)	38 (21.1%)	
	Unknown	38 (14.7%)	23 (14.1%)	19 (10.6%)	
AFP status	0–8	80 (30.9%)	38 (23.3%)	35 (19.4%)	< 0.001
	8–200	95 (36.7%)	40 (24.5%)	41 (22.8%)	
	200–400	16 (6.2%)	8 (4.9%)	11 (6.1%)	
	> 400	56 (21.6%)	68 (41.7%)	86 (47.8%)	
	Unknown	12 (4.6%)	9 (5.5%)	7 (3.9%)	
PT, median (IQR)		13.3 (12.7, 14.3)	13.4 (12.6, 14.2)	13.15 (12.5, 14.25)	0.58
INR, median (IQR)		1.11 (1.05, 1.2)	1.12 (1.05, 1.2)	1.1 (1.045, 1.215)	0.86
Tbil, median (IQR)		13.7 (9.6, 19.5)	14 (10, 21.5)	14.1 (9.85, 20.75)	0.58
Dbil, median (IQR)		5.3 (3.7, 8.7)	6.3 (4.1, 10.7)	6.4 (4.5, 10.45)	0.012
albumin, median (IQR)		38 (34.2, 41.7)	36.9 (33.5, 40.1)	35.95 (31.8, 39.4)	< 0.001
AST, median (IQR)		32 (23, 48)	37 (25, 55)	41.5 (26, 59.5)	0.005
ALT, median (IQR)		31 (21, 43)	31 (20, 46)	32.5 (23.5, 49.5)	0.31
ALP, median (IQR)		86 (67, 120)	101 (78, 142)	111 (87.5, 151.5)	< 0.001
ALBI score, median (IQR)		− 2.52 (− 2.83, − 2.10)	− 2.38 (− 2.70, − 2.04)	− 2.28 (− 2.62, − 1.93)	< 0.001
ALBI grade	1	113 (43.6%)	59 (36.2%)	47 (26.1%)	0.002
	2	137 (52.9%)	92 (56.4%)	123 (68.3%)	
	3	9 (3.5%)	12 (7.4%)	10 (5.6%)	
CTP class	A	212 (81.9%)	131 (80.4%)	139 (77.2%)	0.63
	B	45 (17.4%)	30 (18.4%)	37 (20.6%)	
	C	2 (0.8%)	2 (1.2%)	4 (2.2%)	
TD ≥ 42 Gy	No	13 (5.0%)	69 (42.3%)	73 (40.6%)	< 0.001
	Yes	246 (95.0%)	94 (57.7%)	107 (59.4%)	
Fractions	1	6 (2.3%)	0 (0.0%)	0 (0.0%)	< 0.001
	2	6 (2.3%)	1 (0.6%)	0 (0.0%)	
	3	230 (88.8%)	68 (41.7%)	44 (24.4%)	
	4	11 (4.2%)	86 (52.8%)	85 (47.2%)	
	5	6 (2.3%)	7 (4.3%)	47 (26.1%)	
	6	0 (0.0%)	1 (0.6%)	4 (2.2%)	
Per dose, median (IQR)		15 (14, 15)	11.5 (11.125, 13)	10.5 (9, 10.625)	< 0.001
Recurrence/residual disease	No	137 (52.9%)	83 (50.9%)	72 (40.0%)	0.022
	Yes	122 (47.1%)	80 (49.1%)	108 (60.0%)	
BCLC stage	A	139 (53.7%)	45 (27.6%)	30 (16.7%)	< 0.001
	B	57 (22.0%)	42 (25.8%)	39 (21.7%)	
	C	60 (23.2%)	74 (45.4%)	107 (59.4%)	
	D	3 (1.2%)	2 (1.2%)	4 (2.2%)	
Tumor size, median (IQR)		3.7 (2.5, 6)	6 (4, 9.4)	8.1 (5.45, 11)	< 0.001

AFP, alpha fetal protein; ALBI, albumin-bilirubin score; ALT, alanine aminotransferase; AST, aspartate aminotransferase; BCLC, Barcelona Clinic Liver Cancer; HBV, hepatitis B virus; INR, International Normalized Ratio; PT, prothrombin time;

### Clinical effectiveness of increasing radiation dose

The median tumor size was 5.6 cm (interquartile range [IQR] 1.1–21.0 cm). The median follow-up time was 50.0 months (IQR 6–100 months). When RT dose was used to classify the patients into the SaRT, SbRT, and ScRT groups, 3 notably different curves were observed for long-term post-SBRT survival.

**Fig. 1:**
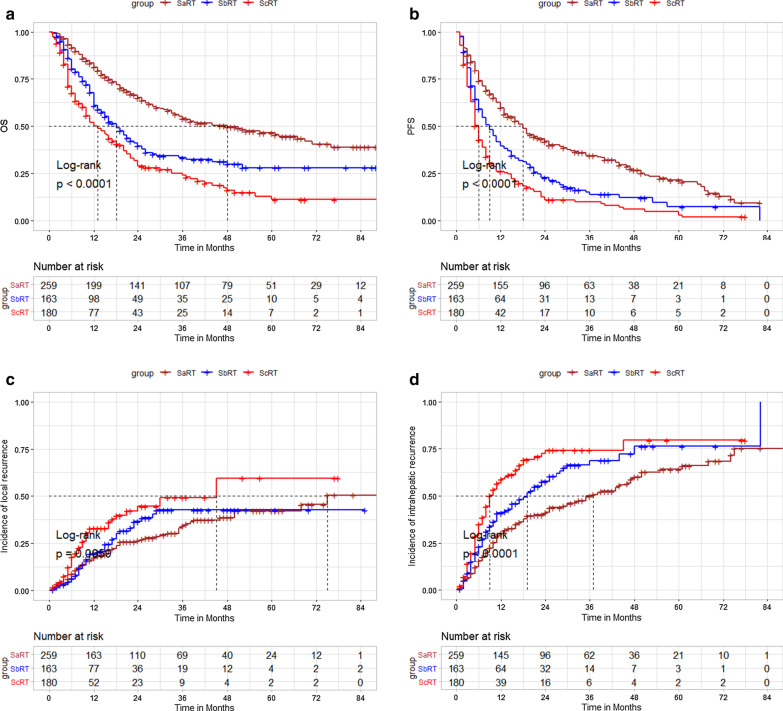
SaRT *versus* SbRT or ScRT: **a** overall survival, **b** progression-free survival, **c** local control, **d** intrahepatic control

The 1-, 2-, 3-, and 5-year OS rates were 81.4, 64.9, 54.1, and 46.4% in the SaRT group; 67.7, 39.5, 33.3, and 28% in the SbRT group; and 50.0, 28.7, 24.0, and 11.1% in the ScRT group, respectively (log-rank *P* < 0.0001; Fig. 1a).

The 1-, 2-, 3-, and 5-year PFS rates were 59.6, 41.8, 34.3 and 21.5% in the SaRT group; 39.5, 22.6, 13.8, and 7.2% in the SbRT group; and 22.5, 10.3, 9.3, and 5.2% in the ScRT group, respectively (log-rank *P* < 0.0001; Fig. 1b).

The 1-, 2-, 3-, and 5-year LC rates were 82.5, 73.7, 65.9 and 57.9% in the SaRT group; 80.6, 63.5, 57.5 and 57.5% in the SbRT group; and 67.2, 55.5, 50.9 and 40.7% in the ScRT group, respectively (log-rank *P* = 0.00594; Fig. 1c).

The 1-, 2-, 3-, and 5-year IC rates were 69.7, 58.7, 50.1 and 36.0% in the SaRT group; 59.1, 42.5, 31.4 and 23.9% in the SbRT group; and 41.2, 27.6, 25.9 and 20.7% in the ScRT group, respectively (log-rank *P* < 0.0001; Fig. 1d).

### Multivariable Cox analysis

Cox proportional hazards models accounting for clustering were used to compare the SaRT, SbRT, and ScRT groups. The selection of influencing factors without associations between each other, including: age, gender, hepatitis B virus (HBV) status, AFP, PT, AST, ALT, alkaline phosphatase (ALP), albumin-bilirubin (ALBI) score, RT dose, recurrence/residual disease, Barcelona Clinic Liver Cancer (BCLC) stage, and tumor size, were considered for multivariate analysis based on *P* value < 0.05 in univariable analyses.

**Fig. 2:**
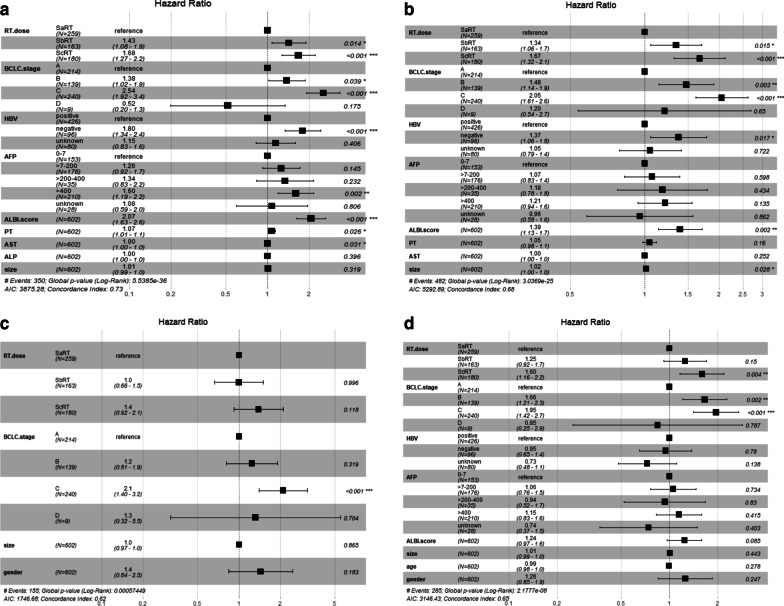
Multivariable cox analyses of all patients. **a** overall survival; **b** progression-free survival; **c** local control; **d** intrahepatic control

Multivariable cox regression analysis of OS (Fig. 2a) showed that 6 independent predictors were RT dosage (SbRT/SaRT: HR = 1.34, 95% CI 1.06–1.7; *P* = 0.015; ScRT/SaRT: HR = 1.67, 95% CI 1.32–2.1; *P* < 0.001), ALBI score, BCLC stage, HBV, AST, and AFP level > 400.

Multivariable cox regression analysis of PFS (Fig. 2b) showed that 5 independent predictors were RT dosage (SbRT/SaRT: HR = 1.43, 95% CI 1.08–1.9; *P* = 0.014; ScRT/SaRT: HR = 1.68, 95% CI 1.27–2.2; *P* < 0.001), ALBI score, BCLC stage, HBV, and tumor size.

Multivariable cox regression analysis of LC (Fig. 2c) showed that BCLC stage was an only independent predictor.

Multivariable cox regression analysis of IC (Fig. 2d) showed that 2 independent predictors were RT dosage (SbRT/SaRT: HR = 1.25, 95% CI 0.92–1.7; *P* = 0.15; ScRT/SaRT: HR = 1.60, 95% CI 1.12–2.2; *P* = 0.004) and BCLC stage.

### Subgroup analysis of total dose and fractionation scheme for OS and PFS

**Fig. 3:**
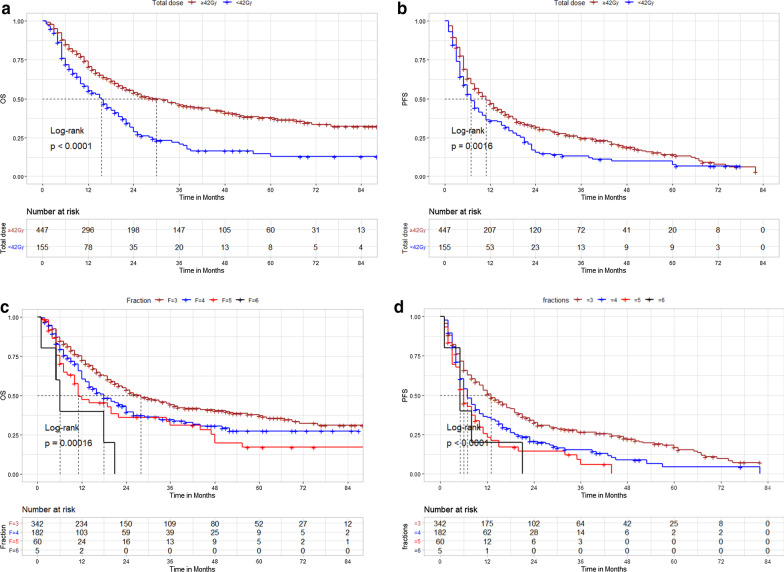
**a** Overall survival: total doses ≥ 42 Gy *versus* < 42 Gy group; **b** progression-free survival: total doses ≥ 42 Gy *versus* < 42 Gy group; **c** overall survival: fractions = 3 *versus* ≥ 4–6 fractions group; **d** progression-free survival: fractions = 3 *versus* ≥ 4–6 fractions group

Additionally, we found a significant association between higher total dose (TD) and better OS. The 1-, 3-, and 5-year OS rates were 70.6, 46.0, and 37.8% in the TD ≥ 42 Gy group and 55.1, 28.9, and 12.9% in the TD < 42 Gy group, respectively (log-rank *P* < 0.001; Fig. 3a). The 1-, 3-, and 5-year PFS rates were 46.8, 31.7, and 14.0% in the TD ≥ 42 Gy group and 35.8, 17.1, and 7.8% in the TD < 42 Gy group, respectively (log-rank *P* < 0.001; Fig. 3b).Further, patients treated with single fraction (n = 6) and 2 fractions group (n = 7) were excluded (Additional file 1: Supplementary Fig. S1), we found that the use of fewer fractions (= 3 fractions) group was association with significantly better OS (Fig. 3c) and PFS (Fig. 3d) than more fractions (≥ 4 to 6 fractions) group.

Additionally, a sensitivity analysis excluding the initial 127 overlapping patients [22], three notably different curves were also obtained for long-term post-SBRT survival for log-rank testing among the high, moderate and low dose groups (Additional file 1: Supplementary material Figure S2–3).

## Discussion

Precise SBRT dose is important but uncertain, especially in HCC that can be treated with radical radiotherapy, because primary HCCs tend to be associated with different tolerated liver volumes and degrees of cirrhosis. In the current study, we classified radiotherapy doses into high dose, moderate dose, and low dose levels: SaRT (BED_10_ ≥ 100 Gy), SbRT (EQD_2_ > 74 Gy to BED_10_ < 100 Gy), and ScRT (EQD_2_ < 74 Gy). Three notably different curves were obtained for long-term post-SBRT survival and intrahepatic control. On multivariate analysis, higher RT dose was associated with improved OS, PFS, and intrahepatic control but not local control. This finding is consistent with a previous study of SBRT for 127 patients with HCCs that were > 5 cm [22].

In the current study, a ≥ 42 Gy total dose and 3 fractions were important indices that were associated with clinical curative effect (e.g. in 42 Gy in 3 fractions, BED_10_ = 100.8 Gy and EQD_2_ = 84.0 Gy).Wahl et al. [16] reported that SBRT appears to be a reasonable first-line treatment for inoperable large HCC. They found no significant difference in OS between the SBRT and RFA groups, and also observed that, for tumors sized ≥ 2 cm, SBRT was superior to RFA in terms of freedom from local progression. In contrast, Rajyaguru et al. [26] reported 5-year OS rates of 19.3% in the SBRT group and 29.8% in the RFA group, and 60 of the 235 (26%) received lower radiation doses (< 40 Gy) in SBRT group, a follow-up analysis of patients receiving ablative doses (> 40 Gy) showed no OS difference in comparison with patients receiving RFA [27]. Jang et al. [28] reported SBRT doses escalated from 33 Gy in 3 fractions to 60 Gy in 3 fractions for HCC (longest diameter ≤ 7 cm). The 2-year OS rates for patients treated with doses > 54 Gy, 45–54 Gy, and < 45 Gy were 71%, 64%, and 30%, respectively, while the 2-year local control rates were 100%, 78%, and 64%, respectively. Recently, a fractionated scheme of 45 Gy in 3 fractions (BED_10_ = 112.5 Gy and EQD_2_ = 93.8 Gy) was tested in a multi-institutional, single-arm phase II trial of SBRT for the treatment of 74 HCC patients with unifocal liver tumors within ≤ 5 cm in diameter in China. Thirteen patients presented with grade ≥ 2 hepatic adverse reaction and 8 patients presented with decreased CP classification [29]. Another scheme, involving 3–5 fractions of 39–50 Gy (EQD_2_ = 70.0 Gy to BED_10_ = 112.5 Gy), has recently been tested in our single-institutional phase II trial of SBRT for the treatment of HCC in patients with a total diameter < 10 cm. A first-line ablative dose of SaRT with a BED_10_ ≥ 100 Gy or a second-line radical dose of SbRT with an EQD_2_ ≥ 74 Gy was recommended. Otherwise, palliative irradiation via ScRT with EQD_2_ < 74 Gy was recommended. In this prior prospective study, 85 patients have been previously reported. None case of classic radiation-induced liver disease was observed. Regarding the Child–Pugh (CP) scores following SBRT, 20 (23.5%) and 12 (14.2%) patients suffered Child–Pugh scores CP +  ≥ 1 and ≥ 2, respectively. We further found that pre-CP, V_15_ (the percentage of normal liver volume receiving more than 15 Gy) and VS_10_ (the absolute normal liver volume spared from at least 10 Gy) were optimal predictors for radiation-induced hepatic toxicity (RIHT: CP +  ≥ 1 and ≥ 2) modelling and nomograms based on normal tissue complication probability (NTCP) models were generated [23]. On the basis of these two studies, optimal selection of SBRT dosage and dose-volume constraints for the liver was recommended to balance the pros and cons (Table 2).

**Table 2:** Recommendations for 3–5 fractions SBRT treatment

Dosimetric constraints for normal liver	Radiation dose for GTV
V_15_ < 21.5%, VS_10_ ≥ 621.8 mL	SaRT: BED_10_ ≥ 100 Gy
V_15_ < 33.1%, VS_10_ ≥ 416.2–621.8 mL	SbRT: EQD_2_ ≥ 74 Gy
Without above conditions or Child–Pugh ≥ B7 class	ScRT: EQD_2_ < 74 Gy

GTV, gross tumor volume; V_15_, percentage of normal liver volume receiving more than 15 Gy; VS_10_, absolute normal liver volume spared from at least 10 Gy

The present study has some limitations. First, the calculation of BED_10_ using an α/β ratio of 10 from the linear-quadratic model is controversial, despite being commonly used. BED_10_ can serve as a simple and straightforward means to perform a comparative and effective analysis among a large variety of dose fractionations prescribed. The clinical efficacy of higher BED_10_ values has been fully recognized in the use of SBRT for lung cancer [19–21] and live cancer [30, 31]. Conventional radiation dose is difficult to exceed 60–74 Gy in HCC, and we found that EQD_2_ ≥ 74 Gy was the second-line radical dose in BED_10_ < 100 Gy. Second, this study was performed in an area in which hepatitis B is endemic; it is unclear whether the dosimetric findings are applicable to cases of HCC associated with other risk factors.

In conclusion, higher radiotherapy doses were associated with better survival in patients undergoing SBRT for the treatment of HCC. If tolerated by normal tissue, we recommend SaRT with BED_10_ ≥ 100 Gy as the first-line ablative dose or undergoing SbRT with EQD_2_ ≥ 74 Gy as the second-line radical dose. Otherwise, ScRT with EQD_2_ < 74 Gy is recommended as palliative irradiation. Future prospective research is warranted to validate the effects of this treatment regimen.

## Supplementary Information

**Additional file 1.** **Fig S1:** Overall survival based on different fractions. **Fig S2:** A sensitivity analysis excluding the initial 127 overlapping patients,three notably different curves of long-term post-SBRT survival: S2A) OS, S2A) PFS.

## Data Availability

The datasets generated during the current study are not publicly available due to hospital secrets but are available from the corresponding author (Su, sutingshi@163.com) on reasonable request.

## Abbreviations

ALBI: Albumin–bilirubin
ALT: Alanine aminotransferase
AST: Aspartate aminotransferase
BCLC: Barcelona Clinic Liver Cancer
BED: Biologically effective dose
CT: Computed tomography
CTP: Child-Turcotte-Pugh
EQD2: Equivalent dose in 2 Gy fractions
HCC: Hepatocellular carcinoma
HBV: Hepatitis B virus
IC: Intrahepatic control
LC: Local control
MRI: Magnetic resonance imaging
OS: Overall survival
PFS: Progression-free survival
RT: Radiation therapy
SBRT: Stereotactic body radiotherapy
