# Supplementary material for: Optimal stereotactic body radiotherapy dosage for hepatocellular carcinoma: a multicenter study
Source: Radiat Oncol. 2021 Apr 21;16:79. doi: 10.1186/s13014-021-01778-6 (PMC8058965; doi:10.1186/s13014-021-01778-6)
Supplement: Supplementary file 1 — Additional file 1. Fig S1: Overall survival based on different fractions. Fig S2: A sensitivity analysis excluding the initial 127 overlapping patients,three notably different curves of long-term post-SBRT survival: S2A) OS, S2A) PFS. [file 13014_2021_1778_MOESM1_ESM.pptx]

## Slide 1
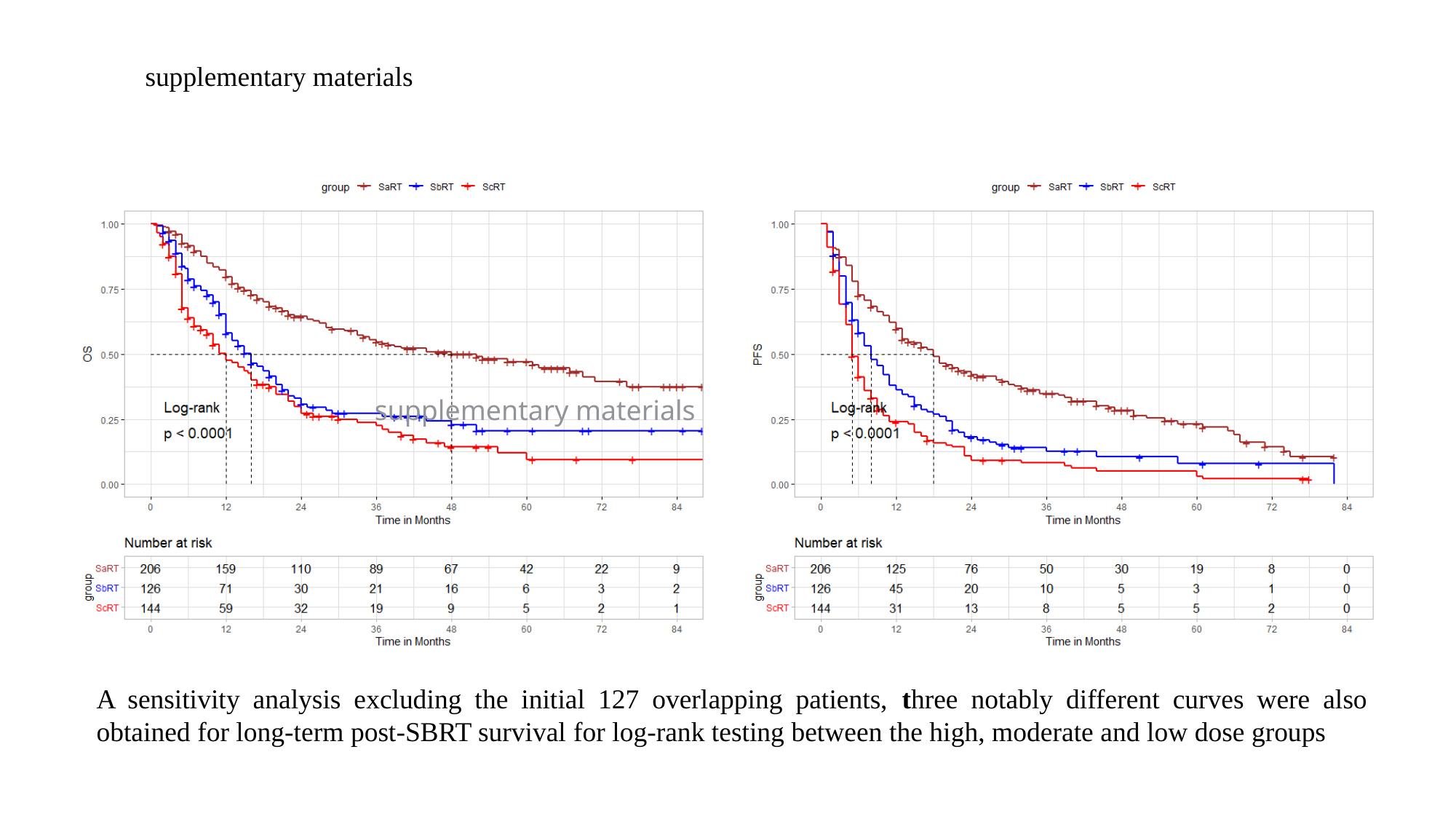

supplementary materials
supplementary materials
A sensitivity analysis excluding the initial 127 overlapping patients, three notably different curves were also obtained for long-term post-SBRT survival for log-rank testing between the high, moderate and low dose groups

## Slide 2
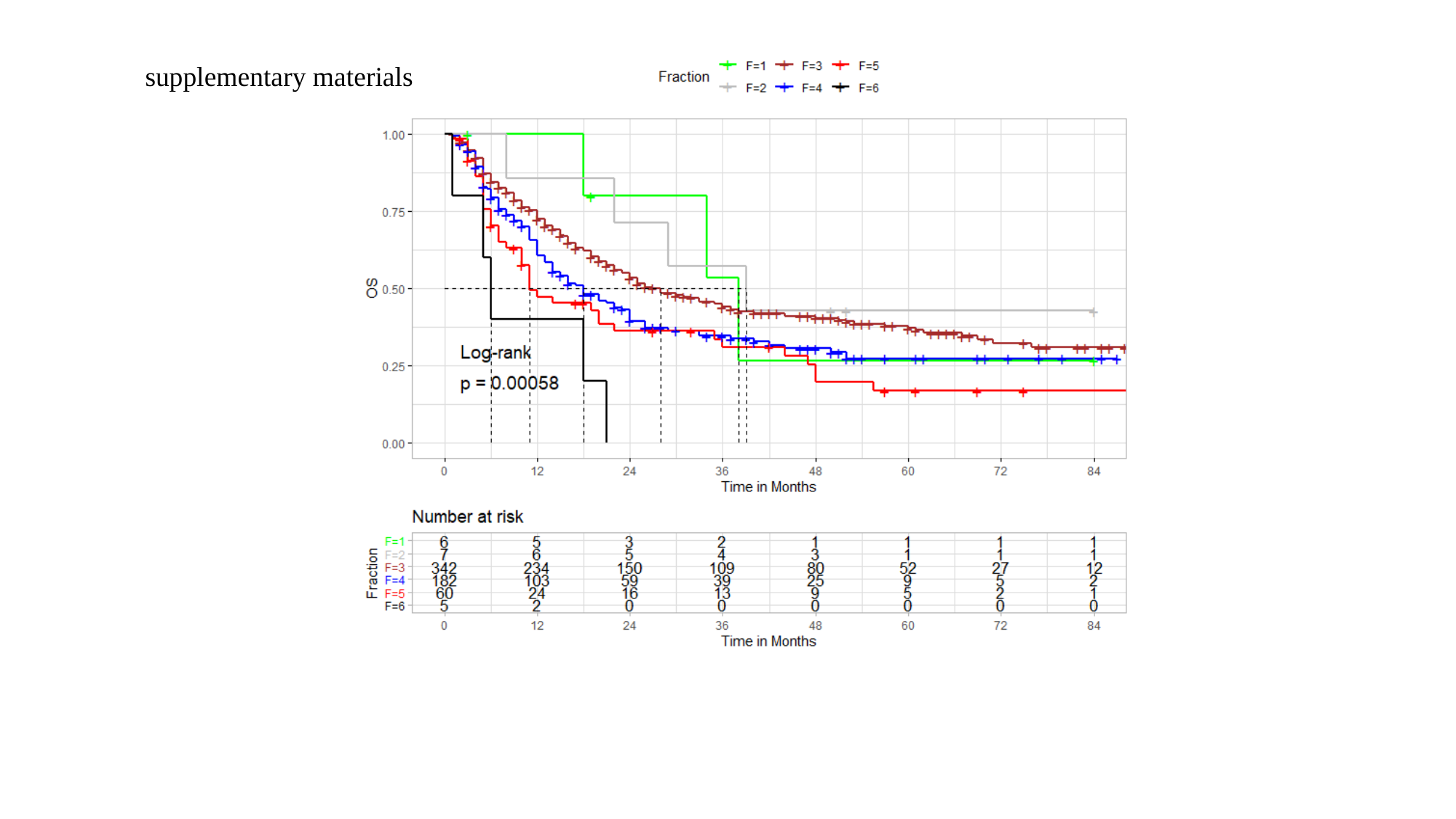

supplementary materials
